# Human N-Alpha-Acetyltransferase 60 Promotes Influenza A Virus Infection by Dampening the Interferon Alpha Signaling

**DOI:** 10.3389/fimmu.2021.771792

**Published:** 2022-01-12

**Authors:** Farjana Ahmed, Matloob Husain

**Affiliations:** Department of Microbiology and Immunology, University of Otago, Dunedin, New Zealand

**Keywords:** N-alpha-acetyltransferase 60, HAT, N-terminal acetylation, influenza virus, interferon α, CH25H, viperin, NAA60 gene

## Abstract

N-alpha-acetyltransferase 60 (NAA60) is the most recently discovered N-terminal acetyltransferase and found only in multicellular eukaryotes. NAA60 localizes to the Golgi complex and is one of the only two N-terminal acetyltransferases known to localize to an organelle. Furthermore, NAA60 possesses a unique ability of catalyzing the acetylation of membrane-anchored proteins at the N-terminus and histones at the lysine side chains. Herein, we demonstrate that NAA60 exhibits proviral properties during influenza A virus (IAV) infection by interfering with the interferon (IFN) α signaling. We found that the depletion and overexpression of NAA60 reduced and enhanced, respectively, the IAV growth in a cell type- and IAV strain-independent manner. Mechanistically, the IAV-induced expression of IFNα was increased and decreased in NAA60-depleted and -overexpressing cells, respectively. Furthermore, the depletion of NAA60 enhanced the level of phosphorylated STAT1 transcription factor as well as the expression of several IFN-stimulated genes (ISGs) such as MX1, CH25H, IFITM3, ISG15 and viperin in infected cells. Whereas the overexpression of NAA60 produced opposite results. Finally, similar results were obtained when the NAA60-depleted cells were treated with purified IFNα. These findings, in conjunction with our recent findings where N-terminal acetylation of many host proteins increased in response to the IAV infection, indicate an important role of N-terminal acetylation during IAV replication.

## Introduction

Influenza A virus (IAV) is the most significant among all known influenza viruses (1). IAV remains a significant threat to global public health as it continues to cause recurring seasonal epidemics, unpredictable pandemics, and zoonotic outbreaks. An RNA genome with segmented nature and a broad host range, including bats (2), contribute to continuous and rapid evolution of IAV and emergence of new variants. This has precluded the development of a universal influenza vaccine and limited the efficacy of annually formulated influenza vaccines. This has also caused the emergence of drug-resistant IAV variants leading to the antiviral drug, adamantanes becoming ineffective (3). Furthermore, the acquisition of several mutations in or around the neuraminidase active site indicates the possibility of the reemergence of the neuraminidase inhibitors-resistant IAV variants (3). Likewise, recently approved polymerase inhibitors may reach the same fate (4). Considering all IAV characteristics and associated challenges, it will be practically impossible to eradicate the IAV from nature. Therefore, there is an undeniable need to continuously study IAV pathogenesis and its interactions with the host to develop effective and alternative antiviral strategies against IAV. One of them is the host-directed antiviral therapies, which may be less prone to the emergence of drug resistance.

To this end, we have been studying the role of acetylation during IAV infection. The acetylation is a protein modification that is now known to exist and be significant in biological processes in all three domains of life (5). Acetylation can occur co- or post-translationally by the addition of an acetyl group reversibly to the lysine or serine side chains or irreversibly to the N-terminal amino group of a polypeptide (6, 7). Two groups of enzymes, acetyltransferases and deacetylases are central to catalyzing and regulating the acetylation level of proteins. The acetyltransferases are divided into three classes: N-terminal acetyltransferases (NATs), histone or lysine acetyltransferases (HATs or KATs) and O-acetyltransferases. Whereas the deacetylases are divided into four classes, of which class I, II, and IV are zinc-dependent histone or lysine deacetylases (HDACs or KDACs) and class III are NAD^+^-dependent sirtuins (6). We have discovered that acetylation promotes IAV infection (8) and at least one member of each class exhibits anti-IAV properties (9–14). Consistent with this, some HATs, e.g., GCN5 and PCAF (15), and NATs, NAA20 and NAA25 (16) have been shown to exhibit pro-IAV properties.

The N-alpha-acetyltransferase 60 (NAA60), also known as NatF or HAT4, is one of the most recently discovered NATs and possesses several unique characteristics. First, NAA60 is a highly conserved enzyme and is found only in multicellular eukaryotes (17). Second, unlike other NATs, NAA60 is localized to an organelle, the Golgi complex, and maintains Golgi integrity and catalyzes the N-terminal acetylation of membrane-anchored proteins (18, 19). Finally, unlike other NATs, NAA60 also exhibits lysine acetyltransferases activity (20). However, the role of NAA60 has not been investigated during IAV infection. Here, we demonstrate that NAA60 promotes IAV infection through suppressing the interferon-alpha (IFNα) signaling.

## Materials and Methods

### Cells and Plasmids

Human alveolar epithelial cells, A549 and Madin-Darby canine kidney (MDCK) cells were grown in minimum essential medium (MEM) supplemented with 10% foetal bovine serum (FBS), 1% L-glutamine and 1% penicillin-streptomycin (Life Technologies) at 37°C under 5% CO_2_. Human bronchial epithelial cells, HBEC3-KT (ATCC) were grown in HBEC3-KT growth kit (ATCC). Plasmid expressing human NAA60 (kindly gifted by Prof Thomas Arnesen and Dr Henriette Aksnes, University of Bergen, Norway) was amplified in *Escherichia coli* DH5α cells and extracted using a plasmid purification kit (Qiagen).

### Viruses and Infection

Influenza virus A/Puerto Rico/8/1934/(H1N1) and A/California/07/2009/(H1N1) were propagated in 10-day old embryonated chicken eggs and titrated on MDCK cells. For infection, cell monolayers grown in individual wells of a cell culture plate were washed twice with serum-free MEM and incubated with virus inoculum (prepared in serum-free MEM) for 1 hour at 35°C. Following incubation, the inoculum was removed, cells were washed once with serum-free MEM and replaced with fresh serum-free MEM. This point was considered as ‘0 hour’ infection timepoint, and the cells were incubated back at 35°C for desired hours before further processing.

### NAA60 Depletion

Pre-designed human NAA60 gene-targeting small-interfering ribonucleic acid (siRNA) (5’-CCA AGA GUG GCA UCG AGU A-3’) and a non-targeting MISSION^®^ control siRNA were purchased from Sigma-Aldrich. The siRNA was delivered to cells by reverse transfection using Lipofectamine RNAiMax (Invitrogen). For this, 10 nM siRNA and 2 µL RNAiMax were diluted separately in Opti-MEM (Gibco) and incubated for 5 minutes at room temperature. The solutions were then mixed and incubated for further 30 minutes at room temperature. Finally, the siRNA-RNAiMax complex was mixed with cell suspension and cells were seeded in a cell culture plate. The cells were incubated at 37°C for 72 hours before further processing.

### NAA60 Overexpression

The plasmid expressing human NAA60 was delivered to cells by reverse transfection using Lipofectamine 2000 reagent (Invitrogen). For this, 1 µg plasmid DNA and 3 µL Lipofectamine 2000 were diluted separately in Opti-MEM and incubated for 5 minutes at room temperature. The solutions were then mixed and incubated for further 30 minutes at room temperature. Finally, the DNA-Lipofectamine 2000 complex was mixed with cell suspension and cells were seeded in a cell culture plate. The cells were incubated at 37°C for 24 hours before further processing.

### Reverse Transcription Quantitative Real-Time PCR

Total RNA was extracted from cells by using Nucleospin RNA isolation kit (Macherey-Nagel) and used as template to synthesize the cDNA by using PrimeScript RT reagent kit (Takara) by following the manufacturers’ instructions. The reverse transcription quantitative real-time PCR (RT-qPCR) was performed by using pre-designed KiCqStart primers or custom-designed primers (Sigma-Aldrich) and SYBR select master mix (Applied Biosystems) on ViiA7 Real-time PCR system (Applied biosystems). The custom-designed primers for β-actin were: Forward 5′-GACGACATGGAGAAAATCTG-3’, Reverse 5′-ATGATCTGGGTCATCTTCTC-3′. The actin mRNA level was used as a reference and the fold or percent change in the mRNA level of target genes was measured using standard 2^−ΔΔCT^ method.

### Western Blotting

Cells were lysed in a lysis buffer (150 mM NaCl, 50mM Tris-HCl [pH 7.4], 0.5% sodium dodecyl sulfate, 0.5% sodium deoxycholate, 1% Triton X-100 and protease inhibitor cocktail [Roche]), and total protein was estimated using BCA protein assay kit (Pierce) as per the manufacturers’ instructions. Equal protein amounts were resolved by sodium dodecyl sulphate–polyacrylamide gel electrophoresis (SDS-PAGE) along with SeeBlue Plus2 pre-stained protein standards (Invitrogen). The protein was transferred to Protran nitrocellulose membrane (Amersham), which was then blocked with 5% milk or 3% bovine serum albumin (BSA) diluted in tris-buffered saline (TBS). The membrane was probed with primary antibodies: rabbit anti-NAA60 (1:1000; HPA040916, Sigma-Aldrich), rabbit anti-GFP (1:1000; #632592, Takara), mouse anti-NP (1:5000; EMD Millipore or 1:10000; NR-19868, obtained through BEI resources, NIAID, NIH), goat anti-NP (1:10000; kindly provided by Richard Webby, St Jude Children’s Research Hospital, USA), mouse anti-pSTAT1 (pY701, 1:1000; clone 14/P-STAT1; BD Biosciences), mouse anti-STAT1 (1:1000; clone 42/STAT1; BD Biosciences), mouse anti-viperin (1:1000; clone D5T2X; Cell Signaling), rabbit anti-ISG15 (1:1000; Cell Signaling), rabbit anti-IFITM3 (1:1000; Abcam), rabbit anti-acetyl histone H4-Lys8 (1:1000; Cell Signaling), mouse anti-acetyl-lysine (1:1000; AAC03, Cytoskeleton Inc) and rabbit anti-PDI (1:10000; Sigma-Aldrich) antibodies followed by the horseradish peroxidase (HRP)-conjugated anti-mouse, anti-goat, or anti-rabbit (1:5000; Life Technologies or Sigma-Aldrich) IgG antibodies. Some blots were re-probed by stripping with 0.5 M sodium hydroxide for 5-7 minutes at room temperature. The protein bands were visualized with ECL or ECL Prime western blotting detection reagent (Amersham), and the images were acquired on Odyssey Fc imaging system (Li-COR) equipped with Image Studio Lite version 5.2. The images were exported as TIFF and compiled in figures using Adobe Photoshop 2022. If needed, the changes in brightness and/or contrast were applied to whole image. For quantification, the intensity of protein bands was measured in Image Studio Lite software and normalized with the loading control, protein disulfide isomerase (PDI).

### Cell Viability Assay

Cells were incubated with MTT [3-(4,5-dimethylthiazol-2-yl)-2,5-diphenyltetrazolium bromide] reagent (Sigma-Aldrich) for 1 hour at 37°C. Then, dimethyl sulfoxide (DMSO) (Calbiochem) was added to the cells and cells were further incubated for 15 minutes at room temperature on a rocking platform. Then, the absorbance was measured at 570 nm in 680-microplate reader (Bio-Rad).

### Microplaque Assay

The infected cells were harvested in culture medium and centrifuged at 12,000 x g for 1 minute. The supernatant was collected and supplemented with 0.3% BSA (Sigma-Aldrich) and titrated on confluent MDCK cell monolayers by microplaque assay. Briefly, the MDCK monolayers were infected with 10-fold serial dilutions of the culture medium for 1 hour at 35°C. The inoculum was replaced with overlay medium containing 2X serum-free MEM, 0.8% Avicel (RC-185; FMC Biopolymer) and 1 μg/ml TPCK-treated trypsin (Sigma-Aldrich), and cells were incubated for 22-24 hours at 35°C. The overlay medium was removed, and cells were fixed with 4% formalin (Sigma-Aldrich) and permeabilized with 0.5% Triton X-100 in 20 mM glycine. Then, cells were stained with mouse anti-NP (1:1000; EMD Millipore or BEI Resources) followed by the HRP-conjugated anti-mouse IgG antibody (1:1000). The plaques were visualized by TrueBlue substrate (KPL Biosciences).

### Graphs and Statistical Analyses

The graphs were plotted and prepared in Prism 9.0 (GraphPad). Each graph was exported as TIFF and compiled in figures using Adobe Photoshop 2022. The statistical analyses were also performed in the Prism. The *P* values were calculated using student *t* test for two datasets and ANOVA for more than two datasets. When using ANOVA, the Geisser-Greenhouse correction was applied. A *P* value of ≤0.05 was considered significant.

## Results

### NAA60 Promotes IAV Infection

**Figure 1:**
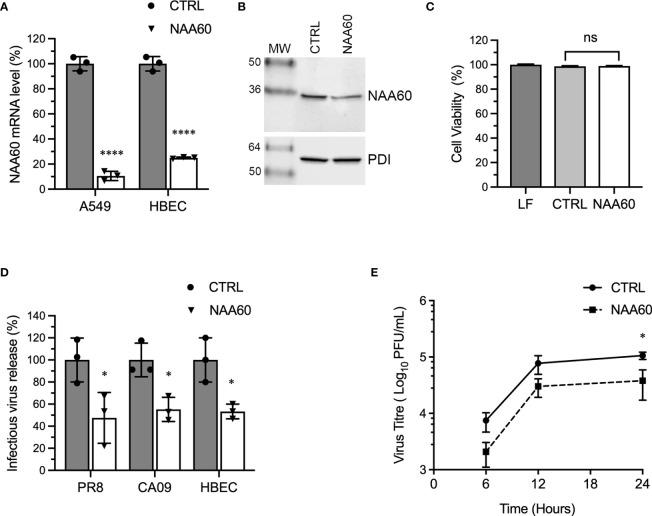
The depletion of NAA60 expression decreases IAV infection. **(A)** A549 cells and HBECs were transfected with 10 nM of control (CTRL) siRNA or NAA60 siRNA for 72 h. Transfected cells were processed to detect the depletion of NAA60 expression by RT-qPCR. The detected NAA60 mRNA level in CTRL and NAA60 siRNA transfected cells were normalized to that of actin, based on which the relative NAA60 mRNA level was calculated by 2^–ΔΔCt^ method. Finally, the NAA60 mRNA level in CTRL siRNA‐transfected cells was considered 100% to determine its level in NAA60 siRNA-transfected cells. **(B)** A549 cells were transfected with 10 nM of CTRL or NAA60 siRNA for 72 h. The cells were processed and the NAA60 (29 kDa) and PDI (57 kDa) polypeptides were detected by western blotting. **(C)** A549 cells were transfected with lipofectamine (LF) only or in a complex with 10 nM CTRL or NAA60 siRNA for 72 h. The viability of cells was then measured by MTT assay. The cell viability in LF treated cells was considered 100% to determine the cell viability in CTRL and NAA60 siRNA transfected cells. **(D)** A549 cells and HBECs were transfected with either CTRL or NAA60 siRNA as above. Then, A549 cells were infected with either PR8 or CA09 and HBECs were infected with PR8 at an MOI of 1.0 for 24 h. The culture medium was collected and titrated by microplaque assay to measure the viral progeny release. The amount of viral progeny released from CTRL siRNA transfected cells was considered 100% to determine the amount of viral progeny released from NAA60 siRNA transfected cells. **(E)** A549 cells, transfected with CTRL or NAA60 siRNA for 72 h, were infected with PR8 at an MOI of 0.1, and the culture medium was collected at 6 h, 12 h and 24 h post-infection and titrated by microplaque assay. Error bars represent means ± standard deviation of at least three biological **(A, D, E)** or technical **(C)** replicates; ****P≤0.0001, *P≤0.05. MW, molecular weight; Grey bar, CTRL; White bar, NAA60; ns, nonsignificant.

We employed RNA interference to investigate the involvement of human NAA60 in IAV infection. For this, we obtained a predesigned siRNA and RT-qPCR primer pair and an antibody for NAA60. There are nine NAA60 transcript variants (generated *via* alternative splicing or promoters) and five polypeptide isoforms in National Center for Biotechnology Information (NCBI; https://www.ncbi.nlm.nih.gov/) and UniProt (https://www.uniprot.org/) database, respectively. The siRNA binds to all NAA60 transcript variants and the primer pair binds to five of them (**Supplementary Figure 1**). The antibody, which has been raised against an N-terminal peptide (Sigma-Aldrich), binds to three isoforms (**Supplementary Figure 1**). The A549 cells and HBECs were transfected with optimized concentration (10 nM) of NAA60 or non-targeting control (CTRL) siRNA, and the depletion of NAA60 was confirmed after 72 hours (h) by RT-qPCR and western blotting. Under these conditions, the NAA60 mRNA was depleted by a significant 89.5% (*P=*<0.0001) and 75.1% (*P=*<0.0001) in A549 cells and HBECs, respectively (**Figure 1A**). Furthermore, the NAA60 polypeptide level was also depleted (**Figure 1B**), by a significant 48.8% (n=3, *P*=0.001); for this, NAA60 polypeptide level in control cells was considered 100% to determine its level in depleted cells. In addition, such depletion of NAA60 reduced the lysine acetylation of histone H4 considerably (**Supplementary Figure 2A**) and other cellular proteins (mostly high molecular weight) slightly (**Supplementary Figure 2B**), without affecting the cell viability (**Figure 1C**). Next, NAA60-depleted and control A549 cells or HBECs were infected with influenza virus A/Puerto Rico/8/1934/(H1N1) (hereafter referred to as PR8) or influenza virus A/California/07/2009/(H1N1) (hereafter referred to as CA09) strains at a multiplicity of infection (MOI) of 1.0. After 24 h, the infectious progeny released in the culture medium was quantified by microplaque assay. We found that, compared to the control, there was a significant 52.5% (*P*=0.04) and 44.8% (*P*=0.014) reduction in the release of PR8 and CA09 infectious progeny, respectively, from NAA60-depleted A549 cells (**Figure 1D**). Similarly, there was a significant 46.7% (*P*=0.019) reduction in the release of infectious PR8 progeny from NAA60-depleted HBECs compared to the control (**Figure 1D**). Next, we assessed IAV growth kinetics in the absence of NAA60 over 24 h. For this, control and NAA60-depleted A549 cells were infected with PR8 at MOI of 0.1. The culture medium was collected at 6 h, 12 h and 24 h of infection and titrated by microplaque assay to measure total viral progeny release. Consistent with above data, the IAV growth kinetics was slower in NAA60-depleted cells than in control cells (**Figure 1E**). The NAA60-depleted cells released 61% and a significant 64% (*P*=0.038, determined by two-way ANOVA Bonferroni’s test) less infectious viral progeny after 12 h and 24 h of infection, respectively, compared to the control cells (**Figure 1E**).

**Figure 2:**
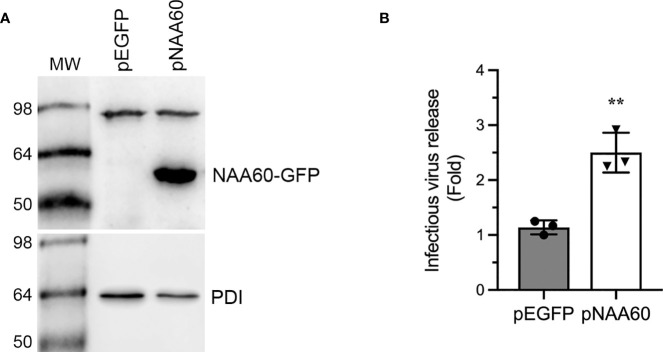
The overexpression of NAA60 increases IAV infection. HEK293T cells were transfected with 1µg of pEGFP or pNAA60 plasmid for 24 h and subsequently infected with PR8 at an MOI of 1.0 for 24 h. **(A)** The cells were processed to detect the NAA60-GFP fusion (56 kDa) and PDI (57 kDa) polypeptides by western blotting. **(B)** The culture medium was titrated by microplaque assay to determine the viral progeny release. The amount of viral progeny released from pEGFP plasmid transfected cells was considered 1-fold to determine the viral progeny released from pNAA60 plasmid transfected cells. Error bars represent means ± standard deviation of at least three biological replicates; **P≤0.01. MW, molecular weight; Grey bar, CTRL; White bar, NAA60.

To support above data, we analyzed the IAV progeny release from the cells overexpressing NAA60. For this, we used HEK-293T cells for their higher transfection efficiency (**Supplementary Figure 3**). HEK-293T cells were transfected with a plasmid (pNAA60) expressing NAA60 as green fluorescent protein (GFP) fusion protein and an empty plasmid (pEGFP) expressing only GFP for 24 h. Then, cells were infected with PR8 at an MOI of 1.0. After 24 h, the culture medium was titrated by microplaque assay and the cells were processed for western blotting to confirm the NAA60 overexpression (**Figure 2A**). Consistent with RNA interference data, the overexpression of NAA60 enhanced the release of infectious IAV progeny release by a significant 2.5-fold (*P*=0.003) when compared to the control (**Figure 2B**). Taken together, these data indicated a pro-IAV role for human NAA60.

### NAA60 Suppresses IAV-Induced IFNα Expression and Downstream Signaling

**Figure 3:**
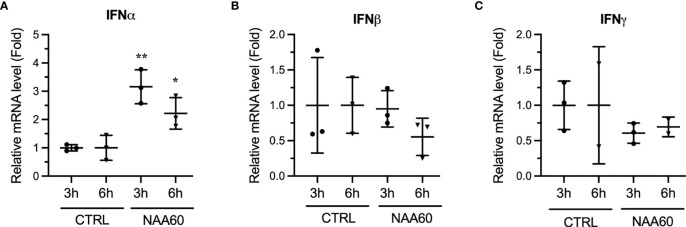
The depletion of NAA60 increases IAV-induced IFNα expression. A549 cells were transfected with 10 nM of CTRL or NAA60 siRNA for 72 h. One set of the cells was processed to confirm the depletion of NAA60 by RT-qPCR as in **Figure 1A** (not shown). The other set of the cells was infected with PR8 at an MOI of 1.0 for 3 h or 6 h, and the cells were processed to detect and calculate the mRNA level of IFNα **(A)**, IFNβ **(B)**, or IFNγ **(C)** by RT-qPCR as in **Figure 1A**. Error bars represent means ± standard deviation of at least three biological replicates; **P≤0.01, *P≤0.05.

To gain insight into the pro-viral mechanism of NAA60, we investigated its role in IAV-induced host innate antiviral response, because, previously, we identified that the HDACs exerts their anti-IAV function *via* this pathway (9–11, 13). Furthermore, the IAV-induced expression of IFNα was downregulated in HDAC11-depleted cells (13) and HDAC6-knockout mice (14). To determine if the opposite was true in NAA60-depleted cells, A549 cells were transfected with the control or NAA60 siRNA and infected with PR8 at an MOI 1.0. After 3 h and 6 h of infection, cells were processed for RT-qPCR to detect the mRNA levels of IFNα, IFNβ, IFNγ and NAA60. Indeed, compared to control, the expression of IFNα mRNA was upregulated in NAA60-depleted cells by a significant 3.2-fold (*P*=0.0036) and 2.2-fold (*P*=0.0413) after 3 h and 6 h of infection, respectively (**Figure 3A**). However, under the same conditions, no significant change was observed in the expression of IFNβ or IFNγ in NAA60-depleted cells (**Figures 3B, C**). This data indicated that NAA60 suppresses the expression of IFNα in IAV-infected cells. The 3 h and 6 h post-infection timepoints were chosen because the transcription of IFNs and IFN-stimulated genes (ISGs) occurs and peaks within few hours of infection and then starts subsiding due to negative feedback mechanism (21, 22). The same is evident here as the expression of IFN mRNAs is either not changing or subsiding from 3 h to 6 h post-infection in control or NAA60-depleted cells. The transcription of different IFNs is temporal and differential in response to virus infection, with IFNα transcription and IFNα-induced transcription of ISGs occurring early during infection compared to IFNβ or IFNγ (21–23). It seems that NAA60 is involved early in the host anti-IAV signaling events mediated by IFNα, not IFNβ or IFNγ.

**Figure 4:**
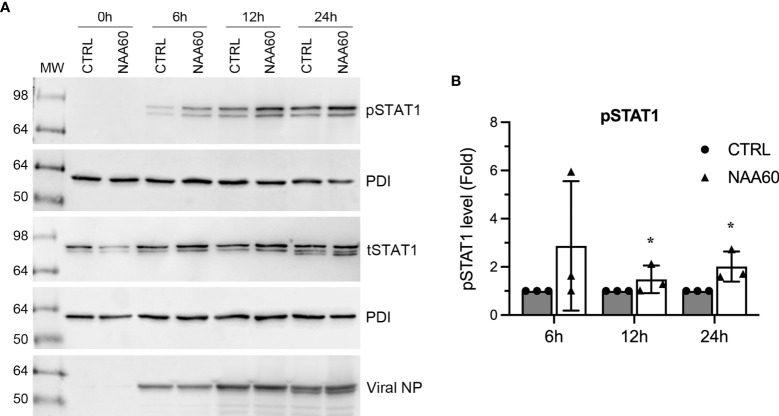
The depletion of NAA60 increases IAV-induced pSTAT1 levels. A549 cells were transfected with 10 nM of CTRL or NAA60 siRNA for 72 h. One set of the cells was processed to confirm the depletion of NAA60 by RT-qPCR as in **Figure 1A** (not shown). The other set of the cells was infected with PR8 at an MOI of 1.0 for 0 h, 6 h, 12 h, or 24 h, and the cells were processed to detect the pSTAT1 (87 kDa), tSTAT1 (87 kDa), viral NP (56 kDa) and PDI polypeptide levels on separate blots by western blotting **(A)**. **(B)** The intensity of pSTAT1, tSTAT1 and corresponding PDI bands at 6 h, 12 h, and 24 h timepoints was quantified. First, the amount of pSTAT1 and tSTAT1 was normalized by the PDI amount at corresponding timepoints. Second, the amount of PDI-normalized pSTAT1 was normalized by the PDI-normalized tSTAT1 amount at each corresponding timepoint. Finally, the double-normalized values of pSTAT1 in CTRL siRNA transfected cells at indicated timepoint was considered 1-fold to determine its amount in NAA60 siRNA transfected cells at the corresponding timepoint. Error bars represent means ± standard deviation of at least three biological replicates; *P≤0.05. MW, molecular weight; Grey bar, CTRL; White bar, NAA60.

One of the consequences of IFNα expression in IAV-infected cells is the phosphorylation-mediated activation of transcription factor, signal transducer and activator of transcription 1 (STAT1) (24). Next, we investigated if the NAA60-mediated suppression of IFNα expression affects STAT1 signaling. For this, A549 cells, transfected with the control or NAA60 siRNA, were infected with PR8 at an MOI of 1.0. Then, cells were harvested after 0 h, 6 h, 12 h and 24 h of infection and the level of the phosphorylated (p) and total (t) STAT1 was detected by western blotting. As expected, in control cells, the pSTAT1 was detected after 6 h of infection and its level gradually increased as the infection progressed (**Figure 4A**) (24). In NAA60-depleted cells, the level of pSTAT1 increased even further after 6 h, 12 h and 24 h of infection compared to controls cells (**Figure 4A**). However, the level of tSTAT1 remained almost steady between control and NAA60-depleted cells after 6 h, 12 h and 24 h of infection (**Figure 4A**). To quantify this change, the intensity of pSTAT1, tSTAT1 and corresponding PDI (loading control) bands from three separate blots (raw images are shown as **Supplementary Figure 4**) was quantified at 6 h, 12 h, and 24 h timepoints. First, the amount of pSTAT1 and tSTAT1 was normalized by PDI amount at corresponding timepoints. Second, the amount of PDI-normalized pSTAT1 was normalized by the PDI-normalized tSTAT1 amount at each corresponding timepoints. Finally, such double-normalized amount of pSTAT1 in control cells at each timepoint (e.g., 12 h) was considered 1-fold to determine its amount in NAA60-depleted cells at the corresponding timepoint (i.e., 12 h). Based on this analysis, we found that the level of pSTAT1 in NAA60-depleted cells after 12 h and 24 h of infection was a significant 1.48-fold (*P*=0.044) and 2.0-fold (*P*=0.048) higher, respectively, than the control cells (**Figure 4B**).

### NAA60 Suppresses IAV-Induced Expression of ISGs

**Figure 5:**
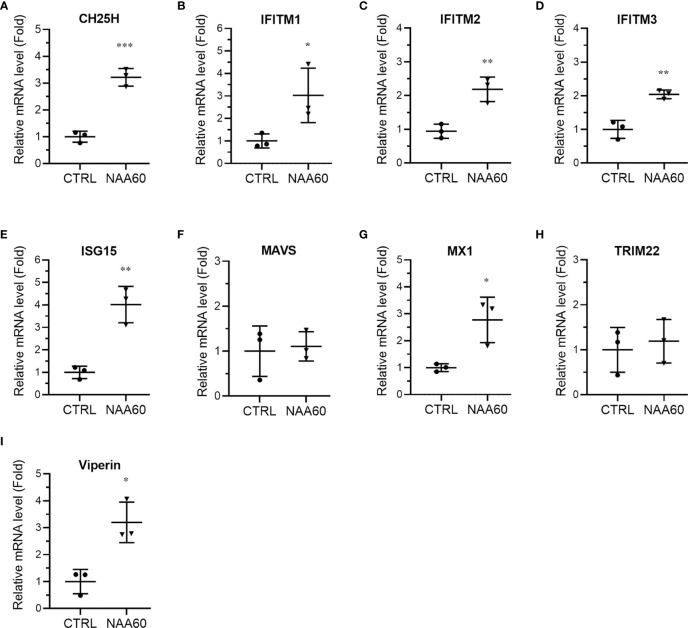
The depletion of NAA60 increases IAV-induced expression of various ISGs. A549 cells were transfected with 10 nM of CTRL or NAA60 siRNA for 72 h. One set of the cells was processed to confirm the depletion of NAA60 by RT-qPCR as in **Figure 1A** (not shown). The other set of the cells was infected with PR8 at an MOI of 1.0 for 3 h, and the cells were processed to detect and calculate the mRNA levels of CH25H **(A)**, IFITM1 **(B)**, IFITM2 **(C)**, IFITM3 **(D)**, ISG15 **(E)**, MAVS **(F)**, MX1 **(G)**, TRIM22 **(H)**, and viperin **(I)** by RT-qPCR as in **Figure 1A**. Error bars represent means ± standard deviation of at least three biological replicates; ***P≤0.001, **P≤0.01, *P≤0.05.

**Figure 6:**
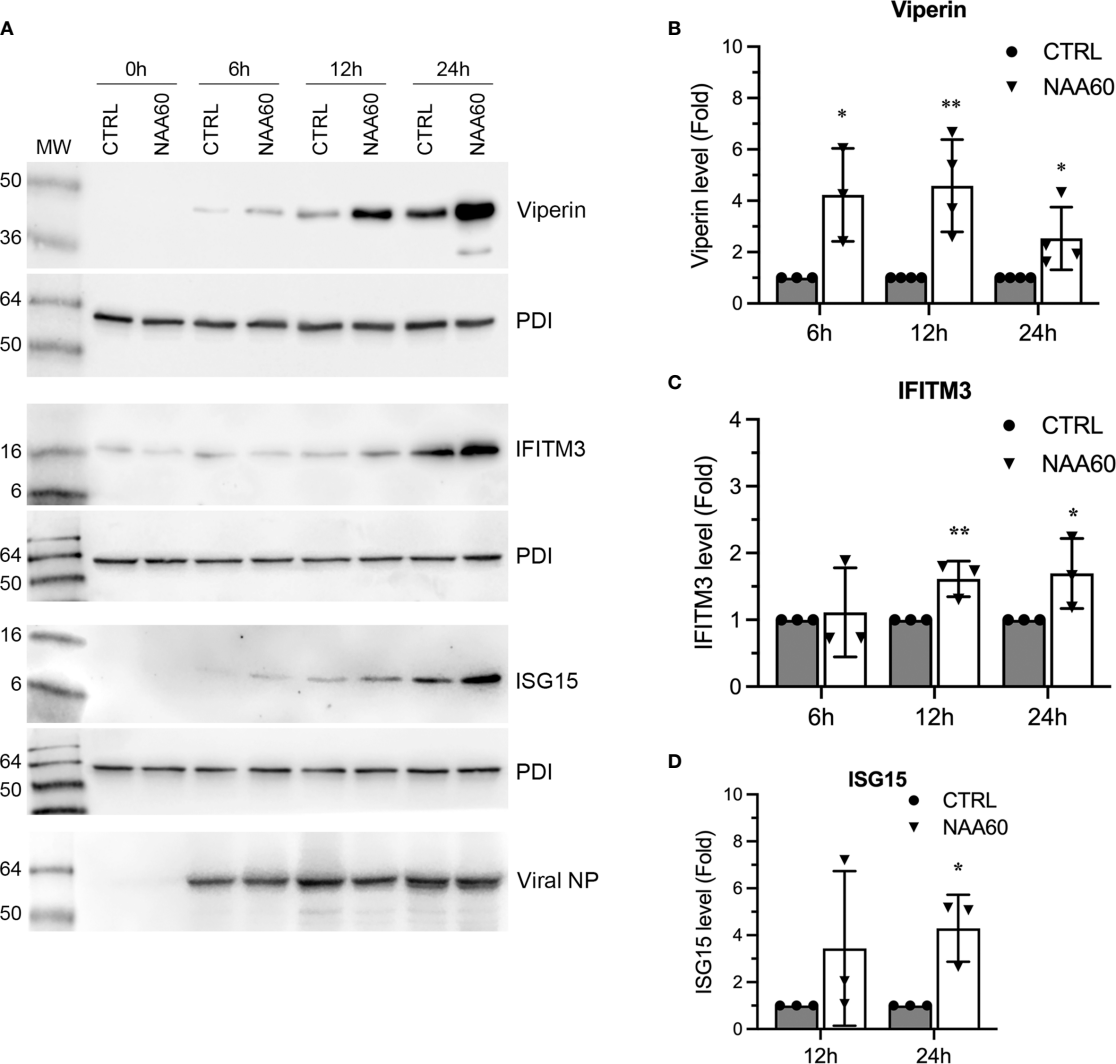
The depletion of NAA60 increases IAV-induced viperin, IFITM3, and ISG15 polypeptide levels. A549 cells were transfected with 10 nM of CTRL or NAA60 siRNA for 72 h. One set of the cells was processed to confirm the depletion of NAA60 by RT-qPCR as in **Figure 1A** (not shown). The other set of the cells was infected with PR8 at an MOI of 1.0 for 0 h, 6 h, 12 h, or 24 h, and the cells were processed to detect the viperin (42 kDa), IFITM3 (15 kDa), ISG15 (15 kDa), viral NP and PDI polypeptide levels by western blotting **(A)**. The intensity of viperin, IFITM3, ISG15 and corresponding PDI bands at 6 h, 12 h, and 24 h timepoints was quantified. Then, the amount of viperin, IFITM3, and ISG15 was normalized by the PDI amount at corresponding timepoints. The normalized values of viperin **(B)**, IFITM3 **(C)**, and ISG15 **(D)** in CTRL siRNA transfected cells at indicated timepoints were considered 1-fold to determine their amounts in NAA60 siRNA transfected cells at corresponding timepoints. Error bars represent means ± standard deviation of at least three biological replicates; **P≤0.01, *P≤0.05. MW, molecular weight; Grey bar, CTRL; White bar, NAA60.

In the next step of IFN signaling, pSTAT1 is translocated to the nucleus and induces the expression of ISGs. Therefore, we next compared the magnitude of the expression of ISGs involved in IAV infection (25), such as cholesterol 25-hydroxylase (CH25H), IFN‐induced transmembrane (IFITM) protein 1, 2 and 3, ISG15, mitochondrial antiviral signaling protein (MAVS), myxovirus resistance protein 1 (MX1), tripartite motif protein 22 (TRIM22), and viperin in control and NAA60-depleted cells in response to the IAV infection. A549 cells were transfected with the siRNAs and infected with PR8 for 3 h, and the mRNA levels of above ISGs were analyzed by RT-qPCR. Consistent with pSTAT1 level, compared to control cells, there was a significant increase in the mRNA levels of CH25H (3.2-fold, *P*=0.0006), IFITM1 (3.0-fold, *P*=0.048), IFITM2 (2.2-fold, *P*=0.006), IFITM3 (2.0-fold, *P*=0.003), ISG15 (4.0-fold, *P*=0.003), MX1 (2.8-fold, *P*=0.022), and viperin (3.2-fold, *P*=0.012) in NAA60-depleted cells in response to IAV infection (**Figures 5A**–**E, G, I**). Whereas no change was evident in the MAVS and TRIM22 mRNA levels between control and NAA60-depleted infected cells (**Figures 5F, H**). Also, no change in the basal mRNA levels of above ISGs (except IFITM2) between control and NAA60-depleted cells was observed without the infection (**Supplementary Figures 5A**–**I**). We also measured the mRNA levels of above ISGs at 6 h post-infection and found a significant increase in the mRNA levels of CH25H (2.3-fold, *P*=0.028) and viperin (2.5-fold, *P*= 0.019) in NAA60-depleted cells (**Supplementarys Figure 6A, I**). However, no significant change was observed in the mRNA levels of other ISGs between control and NAA60-depleted cells (**Supplementary Figures 6B**–**H**). This data seems consistent with the change in IAV-induced IFNα mRNA level between control and NAA60-depleted cells (**Figure 3A**), where increase in the IFNα mRNA level subsided at 6 h post-infection.

Further, we also compared the expression kinetics of viperin, IFITM3 and ISG15 at polypeptide level in control and NAA60-depleted cells in response to IAV infection. These three ISGs have been determined to target three different stages of the IAV life cycle (26–28). For this, A549 cells transfected with the siRNAs and infected with PR8 as above were harvested at 0 h, 6 h, 12 h and 24 h post-infection, and viperin, IFITM3 and ISG15 polypeptides were detected by western blotting. Like their mRNA levels, the polypeptide levels of all three ISGs were further upregulated, albeit at different kinetics, in NAA60-depleted cells (**Figure 6A**). We quantified their upregulation using three separate blots (raw images are shown as **Supplementary Figure 7**) as pSTAT1 above and found that the IAV-induced expression of viperin polypeptide was further enhanced in NAA60-depleted cells by a significant 4.2-fold (*P*=0.014), 4.6-fold (*P*=0.007) and 2.5-fold (*P*=0.045) at 6 h, 12 h and 24 h post-infection, respectively, compared to control cells (**Figure 6B**). Likewise, the IAV-induced expression of IFITM3 polypeptide was further enhanced in NAA60-depleted cells by a significant 1.6-fold (*P*=0.022) and 1.7-fold (*P*=0.041) at 12 h and 24 h post-infection, respectively, compared to control cells (**Figure 6C**). Finally, after 24 h of infection, the IAV-induced expression of ISG15 polypeptide in NAA60-depleted cells was further increased by a significant 4.3-fold (*P*=0.016) compared to control cells (**Figure 6D**).

**Figure 7:**
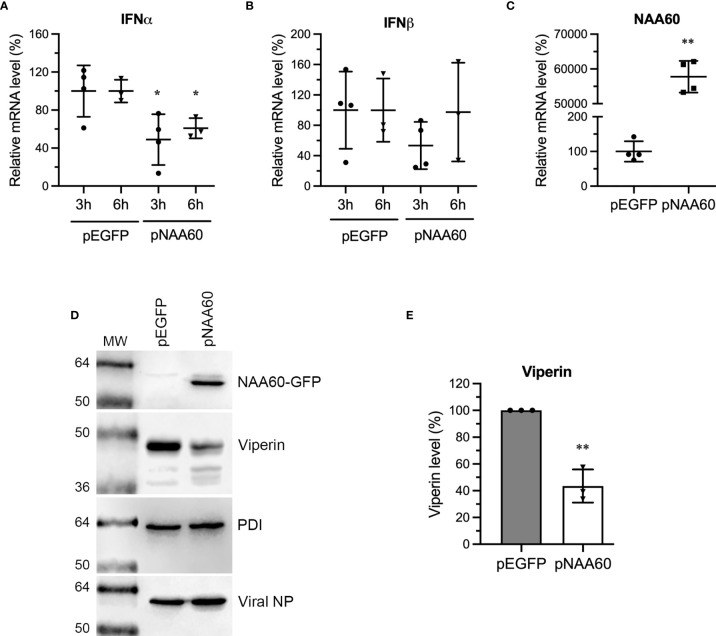
The overexpression of NAA60 decreases IAV-induced IFNα and viperin expression. HEK293T **(A–C)** and A549 **(D, E)** cells were transfected with 1µg of pEGFP or pNAA60 plasmid for 24 h. **(A–C)** One set of the cells was processed to confirm the overexpression of NAA60 by RT-qPCR **(C)** as in **Figure 1A**. The other set of the cells was infected with PR8 at an MOI of 1.0 for 3 h or 6 h, and the cells were processed to detect and calculate the mRNA level of IFNα **(A)** and IFNβ **(B)** by RT-qPCR as in **Figure 1A**. **(D, E)** A549 cells were infected with PR8 at an MOI of 1.0 for 24 h and processed to detect the NAA60-GFP, viperin, viral NP and PDI polypeptide levels by western blotting **(D)**. The intensity of viperin bands was quantified and normalised with the intensity of corresponding PDI bands. The normalized value of viperin in pEGFP transfected cells at each timepoint was considered 100% to determine its amount in pNAA60 transfected cells **(E)**. Error bars represent means ± standard deviation of at least three biological replicates; **P≤0.01, *P≤0.05. MW, molecular weight; Grey bar, CTRL; White bar, NAA60.

Next, we sought to confirm whether ectopic NAA60 expression equally affects the IFNα signaling and produces the results opposite to NAA60 depletion. To do this, HEK293T cells transfected with pEGFP or pNAA60 plasmids as in **Figure 2**, were infected with PR8 at an MOI of 1.0 for 3 h and 6 h. Then, expression of IFNα, IFNβ, and NAA60 mRNA was measured by RT-qPCR (**Figures 7A–C**). Consistent with results in NAA60-depleted cells, the expression of IFNα mRNA in NAA60-overexpressing cells was reduced by a significant 51% (*P*=0.036) and 39.2% (*P*=0.013) at 3 h and 6 h post-infection, respectively, when compared to control cells (**Figure 7A**). Further, no significant change was observed in IFNβ expression (**Figure 7B**). In HEK293T cells, we failed to detect the expression of viperin, IFITM3, or ISG15 polypeptides in response to IAV infection. Hence, we revert to A549 cells, and transfected them with pEGFP or pNAA60 plasmid and infected them with PR8 at an MOI of 1.0 for 24 h and analyzed the expression of viperin polypeptide by western blotting. Opposite to its expression in NAA60-depleted cells, the expression of viperin polypeptide in NAA60-overexpressing cells was decreased (**Figure 7D**), by a significant 56.5% (*P*=0.001) when compared to GFP-expressing cells (**Figure 7E**).

### NAA60 Suppresses IFNα-Induced Expression of IFITM3, ISG15, and Viperin

**Figure 8:**
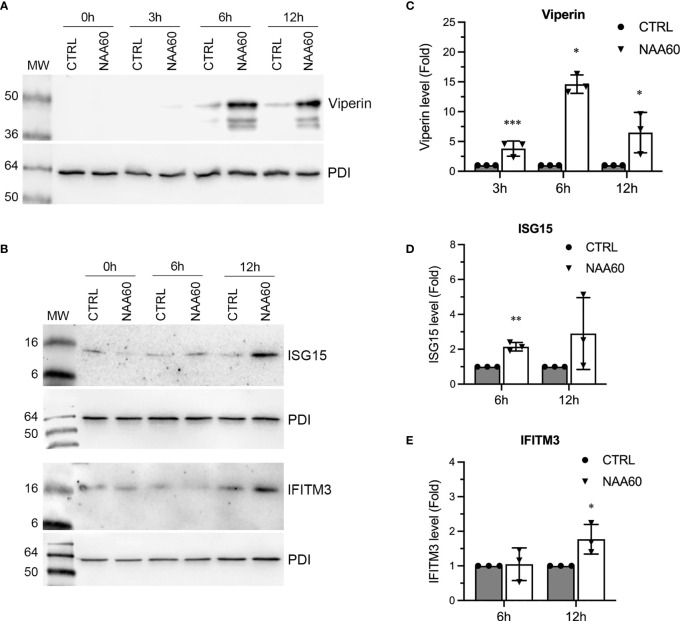
The depletion of NAA60 increases IFNα-induced viperin, IFITM3, and ISG15 polypeptide levels. A549 cells were transfected with 10 nM of CTRL or NAA60 siRNA for 72 h. One set of the cells was processed to confirm the depletion of NAA60 by RT-qPCR as in **Figure 1A** (not shown). The other set of the cells was treated with IFNα at 10 IU/ml for 0 h, 3 h, 6 h, or 12 h, and the cells were processed to detect the viperin **(A)**, IFITM3 and ISG15 **(B)** and PDI polypeptide levels by western blotting. The intensity of viperin, IFITM3 and PDI bands was quantified and normalised with the intensity of corresponding PDI bands. The normalized values of viperin **(C)**, ISG15 **(D)** and IFITM3 **(E)** in CTRL siRNA transfected cells at indicated timepoints were considered 1-fold to determine their amounts in NAA60 siRNA transfected cells at respective timepoints. Error bars represent means ± standard deviation of at least three biological replicates; **P≤0.01, *P≤0.05. MW, molecular weight; Grey bar, CTRL; White bar, NAA60.

The data presented above strongly indicated that NAA60 is involved in IAV-induced IFNα signaling. We sought to further confirm these observations in IFNα-treated cells (in the absence of IAV infection). For this, we transfected A549 cells with control and NAA60 siRNA for 72 h as above, and then treated them with purified IFNα (Human Recombinant Alpha 2a, NR–3083; BEI Resources, NIAID, NIH) at 10 IU/mL for 0 h, 3 h, 6 h, and 12 h. Subsequently, the level of ISG15, IFITM3, and viperin polypeptides was analyzed by western blotting. Like IAV infection, the IFNα treatment also further enhanced the expression kinetics of viperin (**Figure 8A**) and ISG15 and IFITM3 (**Figure 8B**) in NAA60-depleted cells. When quantified, compared to control cells, the level of viperin polypeptide was increased by a significant 3.8-fold (*P*=0.0006), 14.7-fold (*P*=0.011), and 6.6-fold (*P*=0.039) after 3 h, 6 h, and 12 h of IFNα treatment, respectively, in NAA60-depleted cells (**Figure 8C**). Similarly, compared to control cells, the level of ISG15 (**Figure 8D**) and IFITM3 (**Figure 8E**) polypeptides in NAA60-depleted cells was increased by a significant 2.0-fold (*P*=0.001) and 1.72-fold (*P*=0.035) after 6 h and 12 h of IFNα treatment, respectively.

## Discussion

The data presented here demonstrate that host N-terminal acetyltransferase, NAA60 promotes IAV multiplication by interfering with IFNα signaling. We endeavored to support this claim by using in our infection model system, the two IAV H1N1 strains (one lab and one clinical), three human cell lines (one of them bronchial and one alveolar), gene depletion and overexpression, and purified IFNα treatment. However, there seems to be a time lag between the depletion of NAA60 transcript and NAA60 polypeptide, which potentially influences the magnitude of the differences detected here. Further, the effect on IAV infection due to depletion or overexpression of NAA60 could be direct or indirect because various genes and/or cellular pathways could have been affected by the manipulation of NAA60 expression. Nevertheless, our data are consistent with the known proviral role of other acetyltransferases: Gcn5, PCAF (15), p300/CBP (29) and NAA20/25 (16), and complemented by the known antiviral role of multiple deacetylases: HDAC1 (9), HDAC2 (10), HDAC4 (11), HDAC6 (12), HDAC11 (13), and Sirtuins (30) during IAV infection.

Above acetyltransferases and deacetylases exert their proviral and antiviral function, respectively, during IAV infection through both viral and host effectors. The acetyltransferases, Gcn5 and PCAF are involved in the suppression of the expression of host type I IFN and ISGs such as STAT1, MX1 and viperin (15), p300/CBP are implicated in the regulation, both positive and negative, of the expression of multiple host proteins involved in various stages of IAV life cycle (29), whereas NAA20/25 and also Gcn5 and PCAF facilitate the positive regulation of the IAV polymerase and host shutoff activities through acetylation of viral proteins (16, 31, 32). In contrast, HDAC6 (14) and HDAC11 (13) are involved in the enhancement of the IAV-induced type I IFN production. In addition, HDAC 1, 2, 4, and 11 also are implicated in the enhancement of the STAT1 phosphorylation as well as the expression of ISGs like viperin, IFITM3 and ISG15 during IAV infection (9–11, 13). Therefore, the involvement of NAA60 in the suppression of the expression of IFNα and multiple ISGs discovered here to promote IAV infection is consistent with previous findings. Further, this is also consistent with our overall working hypothesis that acetylation-mediated regulation of host innate antiviral response plays a critical part in IAV infection (9–11, 13).

NAA60 may also have additional proviral mechanisms that are distinct from the canonical acetyltransferase functions. NAA60 is only one of the two NATs known to localize to an organelle (NAA60 to Golgi complex and NAA70 to chloroplast), and is the only NAT known to N-terminally acetylate the membrane-anchored proteins post-translationally (7). Among ISGs which were significantly upregulated in NAA60-depleted cells, the CH25H (33) and viperin (34) are membrane-associated proteins and localize to the ER and Golgi complex. CH25H converts cholesterol into more soluble 25-hydroxycholesterol (25HC), which alters the membrane properties. Such alternations interfere with the replication of several enveloped viruses, including of IAV, that particularly rely on the cellular membranes for virus entry, assembly, and release (35, 36). Viperin also targets cholesterol and interferes with its biosynthesis and, consequently, with the replication of multiple enveloped viruses (37). In case of IAV infection, viperin inhibits the release of budding viral progeny from the host cell plasma membrane by interfering with lipid rafts formation (28). Therefore, it is possible that NAA60 regulates the function of CH25H and viperin *via* their N-terminal acetylation in the Golgi complex. Similarly, NAA60 may also regulate the function of Golgi-localized IAV proteins, HA and NA.

The N-terminal acetylation has several consequences for proteins, for example it can: 1) determine their subcellular localization, 2) mediate their interactions with other proteins and promote complex formation, 3) affect their folding and aggregation, and 4) promote their degradation *via* proteasome (7). Recently, we identified 3 IAV proteins (PB1, NS1, NS2) and 53 host proteins to be N-terminally acetylated in infected cells (38). As per the Gene Ontology analysis, those 53 proteins were comprised of both non-membrane and membrane-associated proteins and belonged to different cellular pathways (data not shown). Taken together, the available data indicate that N-terminal acetylation and associated acetyltransferases play a significant role during IAV infection. Further investigations should reveal the influence and significance of this modification on the molecular interplay between IAV and its hosts.

## Data Availability Statement

The original contributions presented in the study are included in the article/**Supplementary Material**. Further inquiries can be directed to the corresponding author.

## Author Contributions

MH conceived the study. FA and MH designed the experiments. FA performed the experiments. FA and MH analysed the data. FA and MH wrote the manuscript. Both authors contributed to the article and approved the submitted version.

## Funding

The H S and J C Anderson Charitable Trust (Dunedin), New Zealand (2018–2020).

## Conflict of Interest

The authors declare that the research was conducted in the absence of any commercial or financial relationships that could be construed as a potential conflict of interest.

## Publisher’s Note

All claims expressed in this article are solely those of the authors and do not necessarily represent those of their affiliated organizations, or those of the publisher, the editors and the reviewers. Any product that may be evaluated in this article, or claim that may be made by its manufacturer, is not guaranteed or endorsed by the publisher.
