# Supplementary material for: Human N-Alpha-Acetyltransferase 60 Promotes Influenza A Virus Infection by Dampening the Interferon Alpha Signaling
Source: Front Immunol. 2022 Jan 12;12:771792. doi: 10.3389/fimmu.2021.771792 (PMC8790067; doi:10.3389/fimmu.2021.771792)
Supplement: Supplementary file 1 [file DataSheet_1.pdf]

Supplementary Material

Human N-alpha-acetyltransferase 60 promotes influenza A virus infection by dampening the interferon alpha signaling

Farjana Ahmed and Matloob Husain*

Department of Microbiology and Immunology, University of Otago, Dunedin 9054, New Zealand

***Correspondence:**

Matloob Husain

matloob.husain@otago.ac.nz

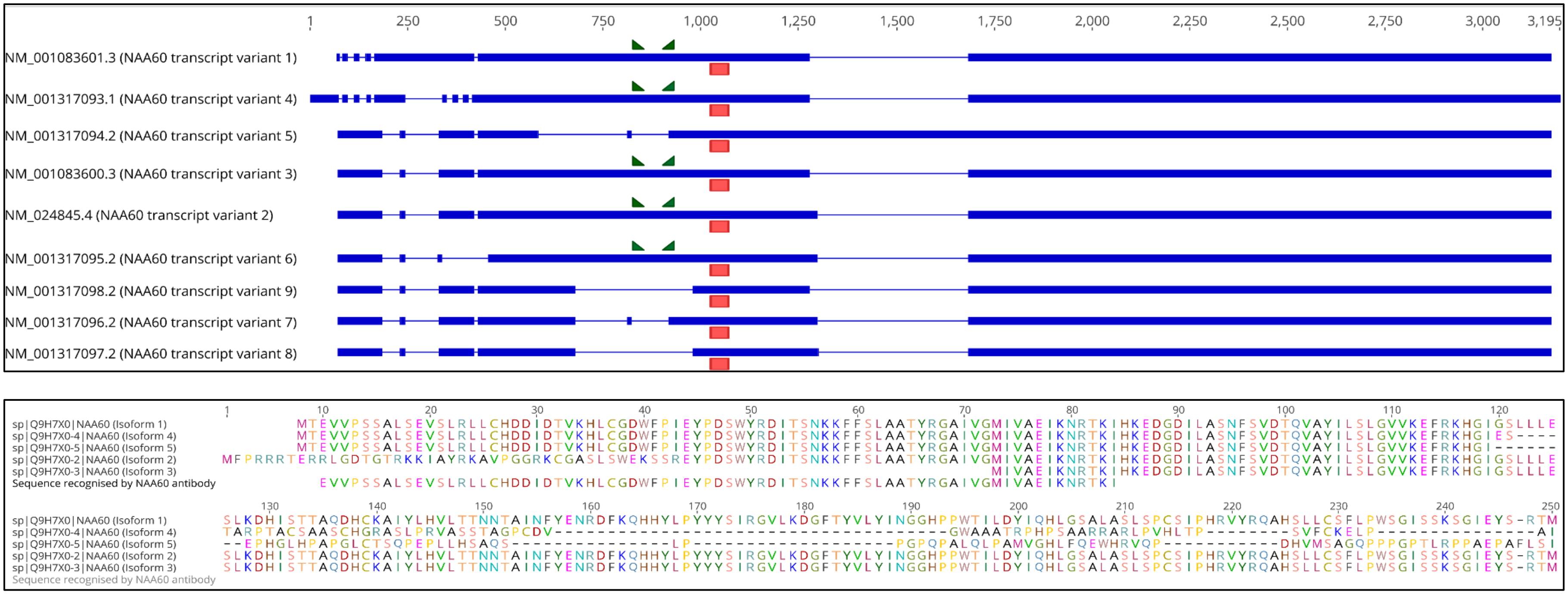

Supplementary Figure 1. Binding profile of NAA60 primer pair (green triangles) and siRNA (red rectangle) to nine NAA60 transcript variants in NCBI database (top box), and NAA60 antibody to five Naa60 polypeptide isoforms in UniProt database (bottom box). Geneious Prime® software using MUSCLE algorithm was used for the alignments.

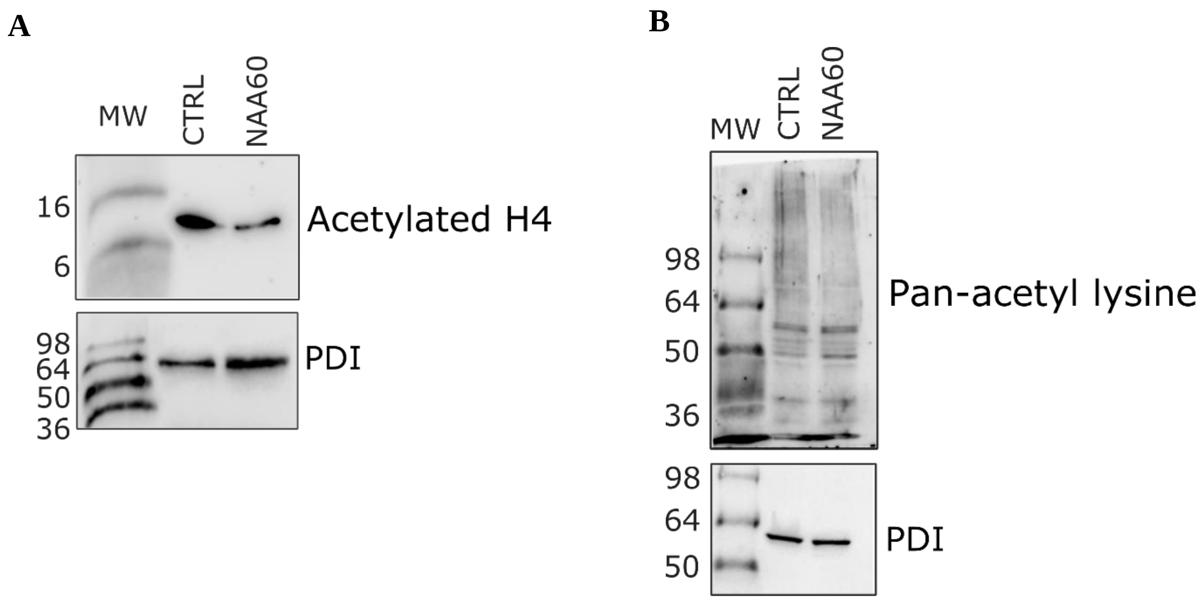

Supplementary Figure 2. The acetylation of histone H4 and high molecular weight proteins was decreased after depletion of NAA60. A549 cells were transfected with 10 nM of control (CTRL) or NAA60 siRNA for 72 hours. Then, total cell lysates were prepared and the level of lysine acetylated histone H4 (11 kDa) **(A)** and other cellular proteins (resolved on 8% SDS-PAGE) **(B)** along with PDI (loading control) (57 kDa) was detected by western blotting. MW, molecular weight.

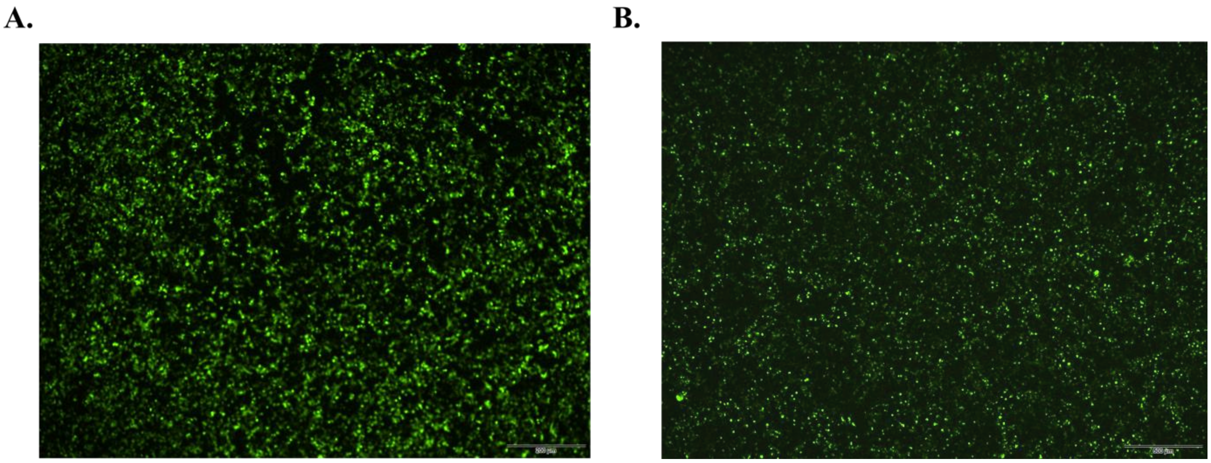

Supplementary Figure 3. The transfection efficiency of pNAA60 plasmid in HEK293T cells. HEK293T cells were transfected with 1 µg of either pEGFP **(A)** or pNAA60 **(B)** plasmid for 24 hours. The cells were then visualized and imaged under an inverted fluorescence microscope (Olympus) at 20X magnification. Scale Bar (at bottom right corner), 200 µm.

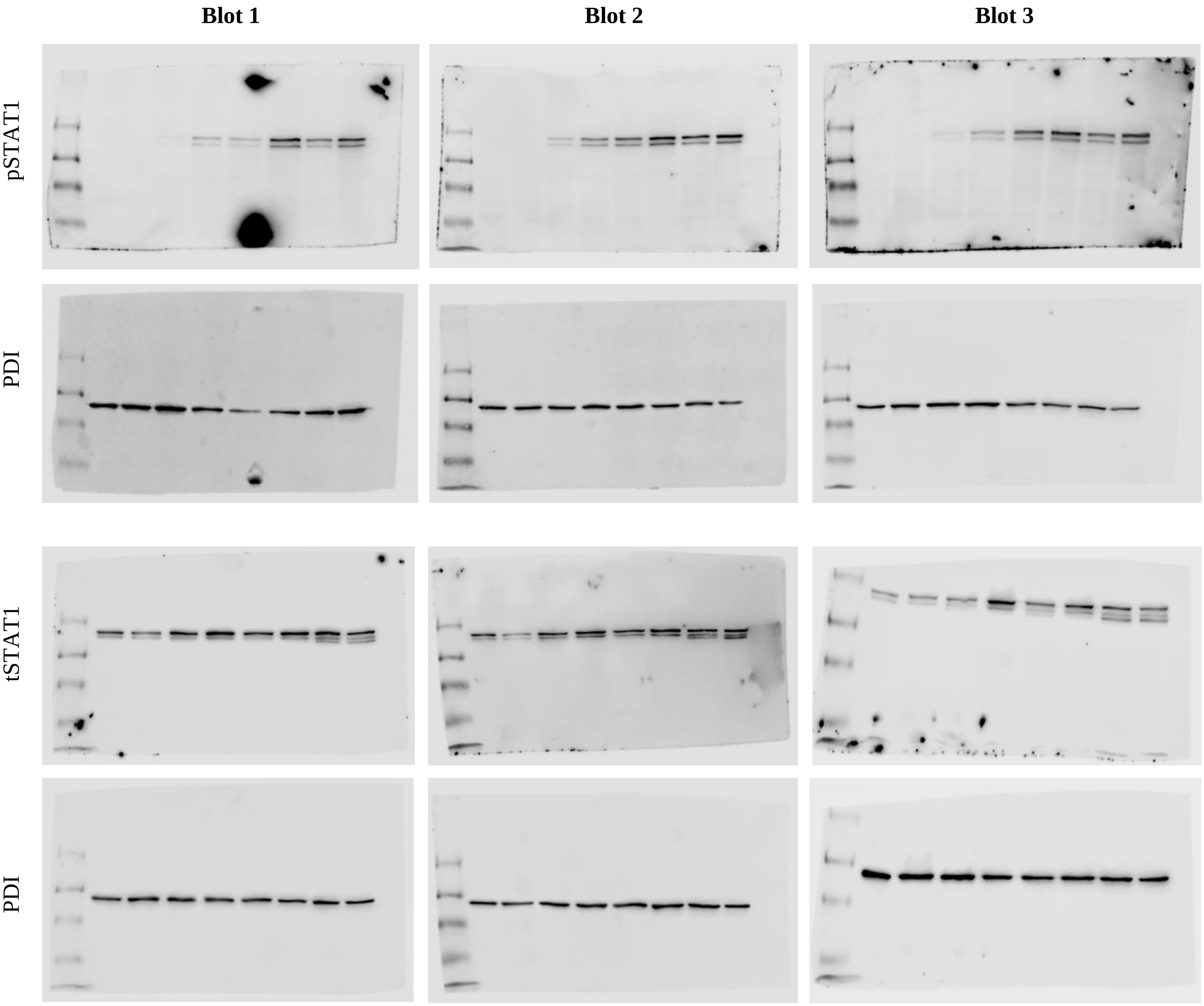

Supplementary Figure 4. Raw images of western blots used for Figure 4B quantification.

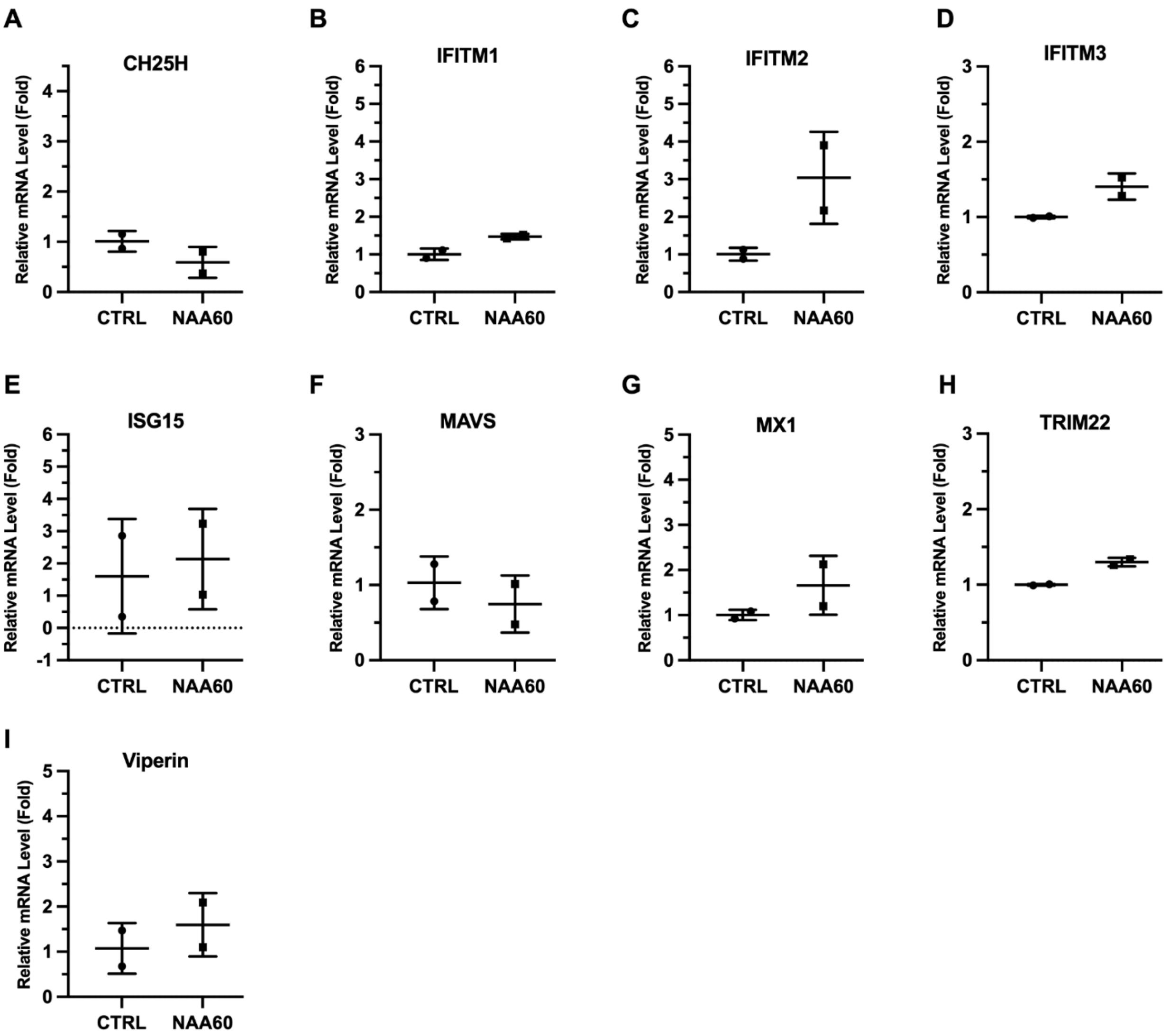

Supplementary Figure 5. The expression of ISG transcripts in NAA60-depleted cells without infection. A549 cells were transfected with 10 nM of CTRL or NAA60 siRNA for 72 hours. The cells were then processed to analyse the mRNA levels of CH25H **(A)**, IFITM1 **(B)**, IFITM2 **(C)**, IFITM3 **(D)**, ISG15 **(E)**, MAVS **(F),** MX1 **(G)**, TRIM22 **(H)**, and Viperin **(I)** by RT-qPCR. Error bars represent means ± standard deviation of two biological replicates.

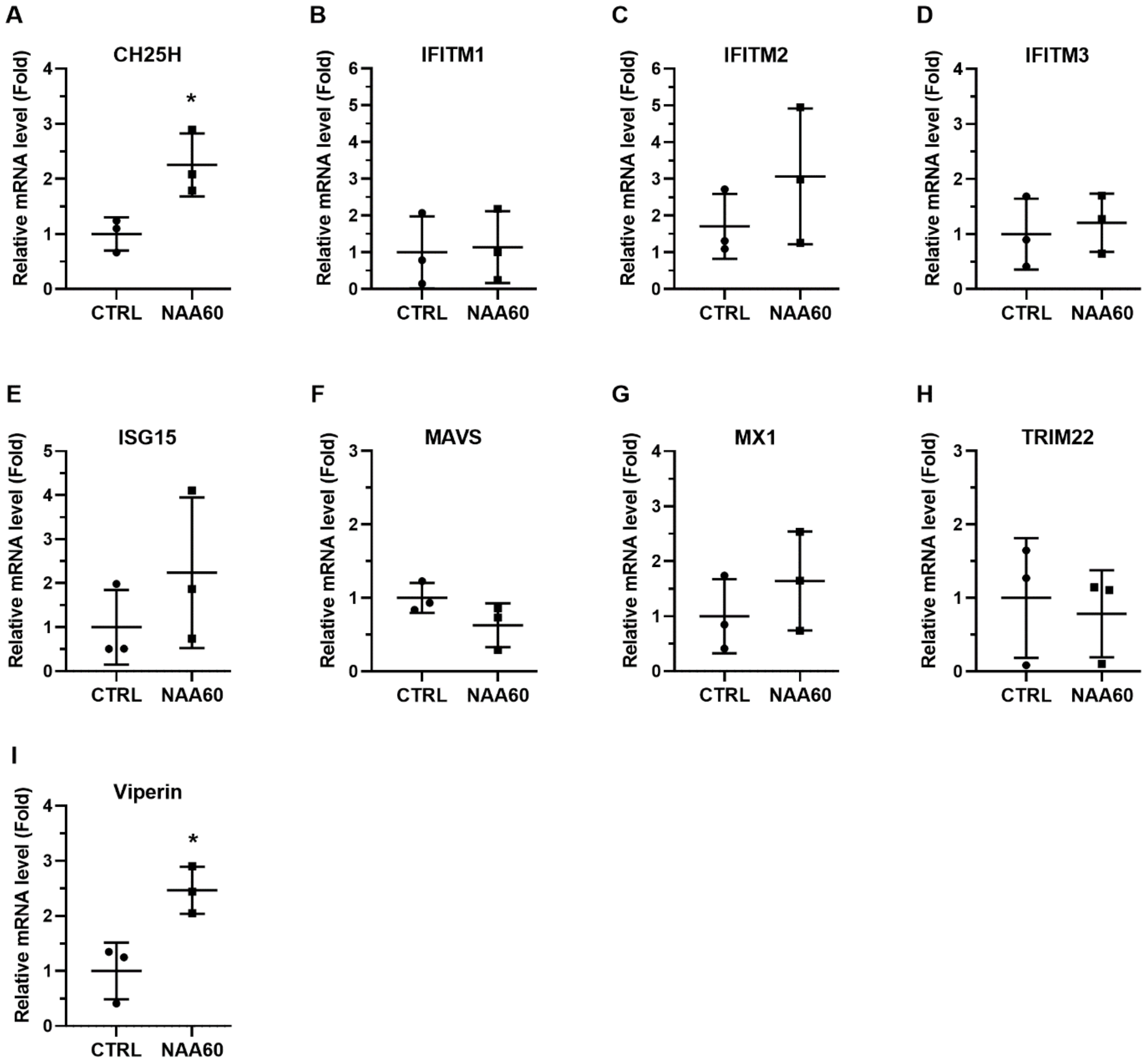

Supplementary Figure 6. The expression of ISG transcripts in NAA60-depleted cells at 6 h post-infection. A549 cells were transfected with 10 nM of CTRL or NAA60 siRNA for 72 hours. One set of the cells were processed to confirm the depletion of NAA60 by RT-qPCR (not shown). The other set of the cells was then infected with PR8 at an MOI of 1.0 for 6 h and the cells were processed to analyse the mRNA levels of CH25H **(A)**, IFITM1 **(B)**, IFITM2 **(C)**, IFITM3 **(D)**, ISG15 **(E)**, MAVS **(F),** MX1 **(G)**, TRIM22 **(H)**, and Viperin **(I)** by RT-qPCR. Error bars represent means ± standard deviation of at least three biological replicates.

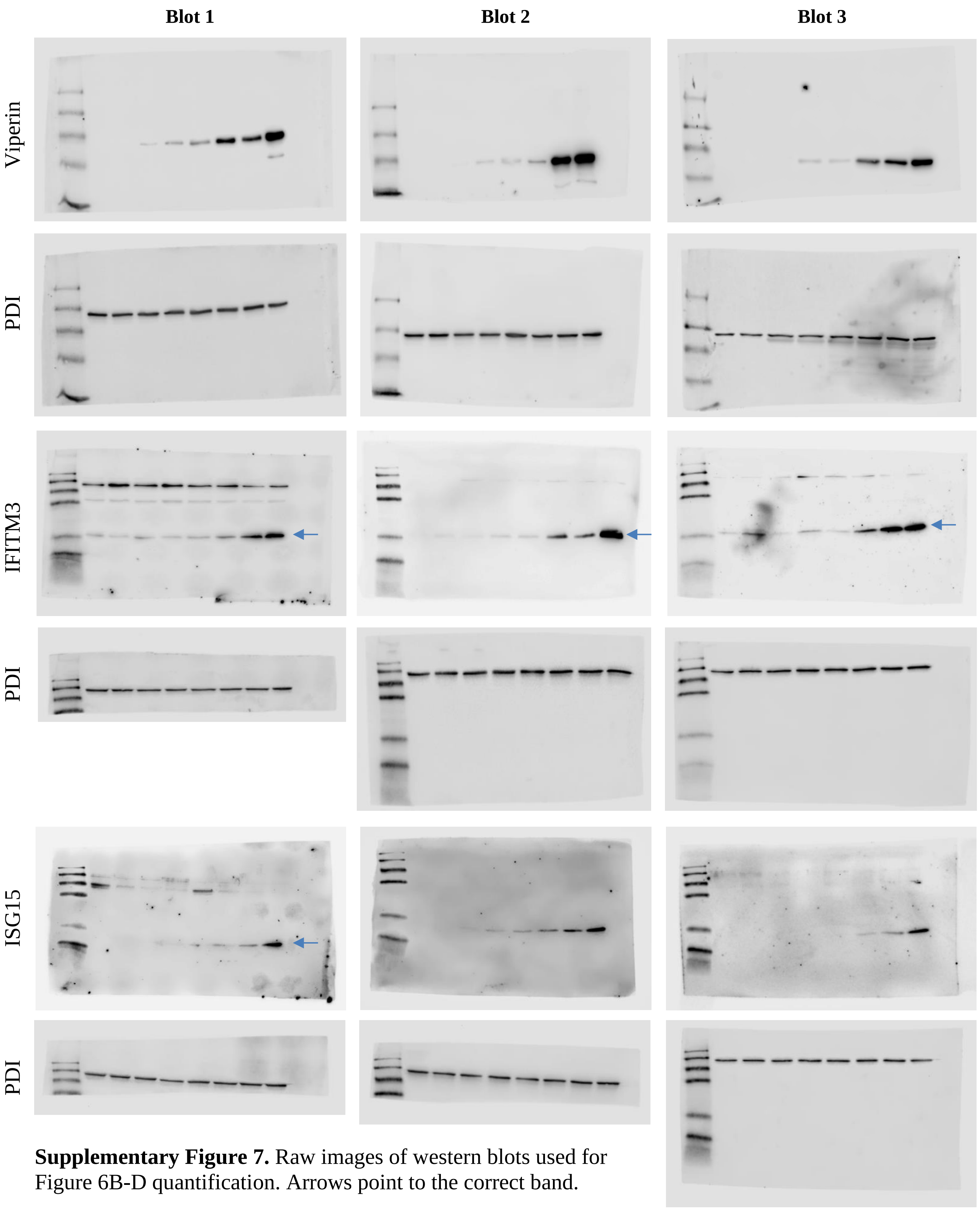

Supplementary Figure 7. Raw images of western blots used for Figure 6B-D quantification. Arrows point to the correct band.
